# Two Cases of Replantation

**Published:** 1904-03-15

**Authors:** J. H. Gibbs


### TWO CASES OF REPLANTATION.

BY J. H. GIBBS, L.D.S.

During a football practice match in February of this year, A. T., aged 28, had his two maxillary central incisors completely dislocated, and the two laterals very much loosened by a knock received from the thumb of an opponent who was trying to collar him. Having come without his teeth, I sent him back to the battlefield to look for them, and they were soon found with the aid of a candle. On examination the teeth were not damaged beyond some earth ingrained into the periodontium, which was pretty completely removed by means of a sharp probe and camel's hair brush. They were placed in weak boric acid solution. Upon examining the mouth, the lips were not injured, but the lateral incisors were extremely loose in their sockets. The socket of the left central was completely destroyed and although the free margin of the gum was intact there was a ragged hole on the labial aspect about a quarter of an inch in diameter extending through the soft parts and bone into the cavity of the socket. The right socket, though considerably damaged, was not nearly so bad. Both centrals were treated alike—pulp cavity opened through the lingual surface, pulp removed, apex closed with sterilized silk impregnated with beta napthol, and the remainder filled with Harvard cement. Each was held in a serviette moistened with boric acid solution. Upon replacing them in the sockets the right tooth remained in place fairly well, but the left one either tumbled into the mouth when let go of or slid through the hole in the gum if pressed home. It being obviously necessary to make a splint which would hold all four teeth in normal relations I took an impression with impression material of the crowns of these and the adjoining teeth, using my left forefinger to keep the root of the left central in position. The centrals came away in the impression and were put in the solution again. A piece of gold plate was struck up and filed so that one edge overlapped the incisal edges for about one-sixteenth of an inch, and the plate extended to the second bicuspid on each side. The plate reached down the lingual surfaces nearly to the gum, and the side against the teeth after polishing was roughened. It was tried in and two holes drilled opposite the neck of each cuspid. Strong silk ligatures, which had been boiled in wax, were passed through the holes and the ends carried through the interspaces on each side of the cuspids, the ligatures being long enough to let the splint rest on the patient's chin. The teeth were put within reach, the surrounding teeth dried as well as possible, cement mixed thin and smeared thickly on the splint. The teeth were replaced, the splint forced firmly into position, and the ligatures at the same time drawn tight and tied round the neck of each canine. The patient was dismissed with directions to use phenol sodique mouth-wash, and to take an aperient. The placing of the splint caused very severe pain in the incisor region which completely subsided within a few minutes. It may be of interest to add that before the patient left he managed to masticate with absolutely no pain, and only the inconvenience of the unaccustomed splint. At his next visit, three days later, everything was satisfactory, and when I next saw him five weeks later he told me that during the fourth week the splint had loosened, a few days later one ligature had given way, and in accordance with instructions he had cut the other and removed the splint. When I saw him the injury to the gum had completely healed, leaving an irregular, depressed scar. The left central was slightly loose, but the others were quite firm, and when I saw him last week all the teeth were quite firm, and he is using them just as freely as ever he did. I should mention, however, that the gum has retracted somewhat from the necks of the central incisors, especially in the interspace, which I am afraid will predispose to caries. Five weeks after the injury I drilled into the lateral incisors and removed the pulp from the left one, and found that that in the right lateral had been already removed, probably ten years ago. There were large gold fillings in all four incisors.

The second case was that of a dental student, who, in the so-called summer session, had a spill from a bicycle whilst going down hill. On picking himself up he found the tooth, and there being fortunately a stream running at the side of the road he washed the tooth, rinsed out his mouth, and replaced the tooth firmly. He was confined to the house for four days, and I saw him on the fifth. On examination the upper lip had been severely cut and three horsehair stitches had been inserted. The left central was very loose, and was discolored and there was a fracture, perhaps only involving the enamel, running horizontally across the tooth, but there was no loss of substance. The left lateral was slightly loose, and a little of the incisal edge had been broken off. The left central, I should say, was, and still is, a trifle extruded from the socket, but is not noticeably so.

An impression was taken of the crowns as before, and a gold splint struck up against the lingual surfaces of the six front teeth and made to overlap the incisal edge about a sixteenth of an inch. It was attached to the teeth in a manner similar to that followed in the first case. A fortnight later the splint loosened, as the mandibular incisors occluded rather heavily with the replaced tooth. Three weeks later the splint was removed, the tooth being then practically fixed. The pulp was removed through a drill hole on the lingual surface and the tooth bleached, being finally filled with cement and gold. The tooth is now quite as firm and as useful as its fellows.—*Dental Record.*

